# Bioelectric modulation of macrophage polarization

**DOI:** 10.1038/srep21044

**Published:** 2016-02-12

**Authors:** Chunmei Li, Michael Levin, David L. Kaplan

**Affiliations:** 1 Department of Biomedical Engineering, Tufts University, 4 Colby Street, Medford, MA 02155; 2 Department of Biology, and Center for Regenerative and Developmental Biology, Tufts University, Suite 4600, 200 Boston Avenue, Medford, MA 02155, USA

## Abstract

Macrophages play a critical role in regulating wound healing and tissue regeneration by changing their polarization state in response to local microenvironmental stimuli. The native roles of polarized macrophages encompass biomaterials and tissue remodeling needs, yet harnessing or directing the polarization response has been largely absent as a potential strategy to exploit in regenerative medicine to date. Recent data have revealed that specific alteration of cells’ resting potential (V_mem_) is a powerful tool to direct proliferation and differentiation in a number of complex tissues, such as limb regeneration, craniofacial patterning and tumorigenesis. In this study, we explored the bioelectric modulation of macrophage polarization by targeting ATP sensitive potassium channels (K_ATP_). Glibenclamide (K_ATP_ blocker) and pinacidil (K_ATP_ opener) treatment not only affect macrophage polarization, but also influence the phenotype of prepolarized macrophages. Furthermore, modulation of cell membrane electrical properties can fine-tune macrophage plasticity. Glibenclamide decreased the secretion and gene expression of selected M1 markers, while pinacidil augmented M1 markers. More interestingly, glibencalmide promoted macrophage alternative activation by enhancing certain M2 markers during M2 polarization. These findings suggest that control of bioelectric properties of macrophages could offer a promising approach to regulate macrophage phenotype as a useful tool in regenerative medicine.

The macrophage polarization refers to development of a specific phenotype in response to local environmental cues^1^,^2^. Functional skewing of macrophage polarization has been involved in physiological conditions (embryogenesis and pregnancy), as well as in pathological conditions (tissue repair, cancer, infection, allergy and chronic inflammation)^3^. As a key determinant of disease development and/or regression, macrophages have emerged as an important therapeutic target in the treatment of many human disease. In response to M1 stimulants (Lipopolysaccharide: LPS, and Interferon-γ: IFN-γ), classically activated macrophages (M1) secrete inflammatory cytokines to kill invading pathogens. Additionally, M1 macrophages produce enzymes to degrade the extracellular matrix. In response to M2 stimuli (Interleukin 4: IL-4 and Interleukin 13: IL-13), wound healing macrophages (M2) produce MMPs (matrix metalloproteinase) and cytokines to promote wound healing and fibrosis. Regulatory macrophages are generated in response to a variety of signals including interleukin 10 (IL-10), apoptotic cells, immune complexes, and glucocorticoids, which secrete high levels of IL-10 to suppress immune responses. The native roles of polarized macrophages encompass the same types of control that biomaterials and tissue remodeling are often sought in tissue repairs and regeneration.

Bioelectric signaling has been shown to be an important regulating mechanism in wound healing and tissue regeneration^4^,^5^,^6^. Studies already demonstrated that human mesenchymal stem cells’ (hMSCs) and tissue level responses to changes in membrane potential significantly modulate cell functions, including differentiation, transdifferentiation and tissue regeneration^7^,^8^. An extensive literature also implicates endogenous bioelectric gradients in the control of wound healing during repair^9^,^10^. Although macrophages are an important regulator of tissue regeneration^11^,^12^ and have been shown to be responsive to external electric fields^13^, very little is as yet known about the bioelectric control of macrophage polarization and how it might affect tissue regeneration.

The goal of this study was to determine whether bioelectric modulation of cell membrane properties would exert control over macrophage polarization. Ion channels play a major role in controlling the membrane potential in lymphocytes and professional antigen presenting cells (APCs), such as monocytes, macrophages, and dendritic cells^14^,^15^. One approach to V_mem_ modulation is to target ion channels expressed on the cell surface with specific channel blocker or openers, which could potentially cause cell depolarization and hyperpolarization, respectively. Immune cells regulate their intracellular Ca^2+^ signaling pathways for proliferation and/or differentiation by modulating the expression and activity of ion channels. A number of potassium (K^+^) channels, including ATP sensitive K^+^ channel (K_ATP_), voltage-gated K^+^ channels (Kv) and Ca^2+^ activated K^+^ channels (KCa), have been detected in macrophages depending on the species, the source of cells, the culture conditions, and their activation and differentiation states^16^,^17^,^18^,^19^. However, the role of K_ATP_ channels in macrophage polarization has never been studied. In this study, we used K_ATP_ channel blocker glibenclamide and opener pinacidil to induce membrane depolarization and hyperpolarization, respectively. The effect of modulation of cell membrane electric properties on macrophage phenotype was investigated using the human monocytic cell line THP-1.

## Methods

### Differentiation and polarization of THP-1 cells into M1 and M2 macrophages

THP-1 cells were obtained from ATCC (Manassas, VA) and maintained in RPMI 1640 medium (Invitrogen, CA) supplemented with 10% heat-inactivated FCS, 100 U/ml penicillin and 100 mg/ml streptomycin. Cells were seeded in 24-well plates at a density of 250,000 cells/well. To generate M1 polarized macrophages, cells were treated with 200 ng/ml phorbol 12-myristate 13-acetate (PMA, EMD) for 6 hours and then cultured with PMA plus 100 ng/ml lipopolysaccharide (LPS, ENZO lifescience, Farmingdale, NY) and 20 ng/ml Interferon-γ (IFN-γ, Peprotech, NJ) for up to 66 hours. To generate M2 polarized macrophages, cells were treated with 200 ng/ml PMA plus 20 ng/ml interleukin-4 (IL-4, Peprotech) and 20 ng interleukin-13 (IL-13, Peprotech) for up to 66 hours. Cultures were maintained at 37 °C in humidified 5% CO_2_ incubator. Cells were tested for mycoplasma contamination and proved to be mycoplasma free.

### Protein extraction and Pathscan ELISA

Cells grown on 10 cm tissue culture dishes were washed once in ice-cold PBS and lysed on ice with ice-cold lysis buffer (20 mM Tris-HCl (pH 7.5), 150 mM NaCl, 1 mM Na_2_ EDTA, 1 mM EGTA, 1% Triton, 2.5 mM sodium pyrophosphate, 1 mM β-glycerophosphate, 1 mM Na_3_VO_4_, and 1 μg/ml leupeptin) supplemented with 1 mM phenylmethylsulfonyl fluoride (PMSF). The cells were then scraped off the plate and sonicated briefly on ice to release the nuclear protein. Sample protein concentration was determined by BCA assay (Pierce, IL). Pathscan ELISA (Cell signaling, MA) was used to determine the phosphorylation of NF-κB p65 (Ser536), Stat1(Tyr701) and Stat6 (Tyr641) according to manufacturer’s instructions.

### Cytokine and chemokine detection by ELISA

Cytokine (TNF-α) and chemokine (CXCL10, CCL22) content in cell culture supernatants was measured using the DuoSet ELISA development kit (R&D Systems, MN) according to manufacturer’s instructions. The assays were run at room temperature. Limits of detection were 15.6 pg/ml for TNF-α, 31.2 pg/ml for CXCL10, and 7.8 pg/ml for CCL22.

### RNA extraction and real time RT-PCR

RNA was isolated and purified using the Qiagen RNEasy kit (Qiagen, CA). The RNA samples were then transcribed into cDNA using High capacity cDNA Archive kit (Applied Biosystems, CA). Real time PCR was performed on cells with and without treatment to track the expression of markers characteristic of the macrophage polarization states. M1 markers include TNF-α and CXCL10, while M2 markers include CD206 and CCL22. The expression of K_ATP_ channel subunits (K_ir_6.1, K_ir_6.2, SUR1 and SUR2) was also determined by PCR. Primers and probes were obtained from Taqman gene expression assay kits (Applied Biosystems, CA). Transcript levels were quantified using the Stratagene Mx3000P QPCR system (Stratagene, CA). PCR reaction conditions were 2 min at 50 °C, 10 min at 95 °C, and then 40 cycles at 95 °C for 15 S, and 1 min at 60 °C. The transcript expression data were normalized to the housekeeping genes, glyceraldehyde-3-phosphate-dehydrogenase (GAPDH).

### Immunocytochemistry of K_ATP_ subunits

Cells were grown on glass-bottom dishes (MatTek Corp., MA) and polarized to M1 and M2 phenotype for 18 hours. The cells were then washed with PBS and fixed with ice-cold 100% methanol. After blocking, cells were labeled with the following primary antibodies: K_ir_6.1 goat polyclonal (Santa Cruz Biotechnology, Santa Cruz, CA), K_ir_6.2 rabbit polyclonal (Abcam, MA), SUR1 rabbit polyclonal (Santa Cruz Biotechnology, TX), SUR2 rabbit polyclonal (Santa Cruz Biotechnology, TX). Alexa Fluor 488 conjugated anti-rabbit and Alexa Fluor 488 conjugated anti-goat IgGs (Molecular probes, OR) were used as secondary antibodies. Samples were mounted in mounting medium (Vectashield Laboratories, CA) and imaged with a Leica TCS SP2 laser scanning confocal microscope with an inverted DM IRE2 stand (Wetzlar, Germany). Samples incubated with secondary antibody alone were used as negative controls.

### Membrane potential modulation

To assess the effect of membrane potential on macrophage polarization, K_ATP_ channel blocker glibenclamide (20 μM, Sigma-Aldrich) and opener pinacidil (100 μM, Sigma-Aldrich) were added to non-stimulated or stimulated macrophages at various time points. In prepolarization treatment study, glibenclamide and pinacidil were added to culture medium 10 minutes before cell stimulation with LPS/IFN-γ (M1) or IL-4/IL-13 (M2). In prepolarized macrophage studies, glibenclamide or piancidil was added to the medium after cells were stimulated with M1 or M2 stimuli for 18 hours. In transdifferentiation studies, cells were stimulated with M1 or M2 stimuli for 18 hours and washed once with PBS. The cells were then treated with glibenclamide or pinacidil for 10 minutes before adding transdifferentiating stimuli (IL-4/IL-13 for M1→2, LPS/IFN-γ for M2→1). Fresh stock solutions of glibenclamide and pinacidil were prepared for each experiment.

### Confocal imaging of membrane potential using DIBAC

Cells grown in glass-bottom were stained with a membrane potential-sensitive dye DiBAC. A stock solution of 10 mM DiBAC in DMSO was prepared and diluted to 5 μM in Hank’s Buffered Salt Solution (HBSS). Cells were incubated at 37 °C for 30 min and imaged while submerged in dye with a Leica TCS SP2 laser scanning confocal microscope with an inverted DM IRE2 stand (Wetzlar, Germany). DiBAC was excited with 488 nm light from a HeNe laser, and the fluorescent light collected at 510–520 nm. The gain and offset settings of the microscope were kept constant over the duration of each experiment for the purpose of quantification of fluorescence intensity. Matlab software was used to calculate the fluorescence intensities of cells by averaging the pixel intensities above preset threshold.

### Fluorescent measurement of membrane potential change using DiSBAC_2_(3)

Measurement of changes of the THP-1 cell membrane potential was performed using a voltage-sensitive dye DiSBAC_2_(3), and the higher cellular uptake of the dye indicates a more depolarized membrane potential. Cells were seeded at a density of 1.5 × 10^6^ cells/well in a 24-well glass-bottom plate (Greiner, NC). After 18 hours of M1 and M2 polarization, DiSBAC_2_(3) was added to culture medium at a final concentration of 100 nM and incubated at 37 °C for 30 minutes. Glibenclamide (20 μM) or pinacidil (100 μM) was then added and the changes in DiSBAC_2_(3) fluorescence intensity was recorded with bottom reading mode using a SpectraMax Paradigm Multi-mode microplate reader (Molecular device, OR).

### Statistical analysis

Statistical analysis was performed by one-way analysis of variance (ANOVA) followed by Bonferroni post-hoc test or by two-tailed Student’s t-test using GraphPad software (GraphPad Prism software, CA). *p* values < 0.05 were considered statistically significant.

## Results and Discussion

### Macrophage polarization model from THP-1 monocytic cells

**Figure 1:**
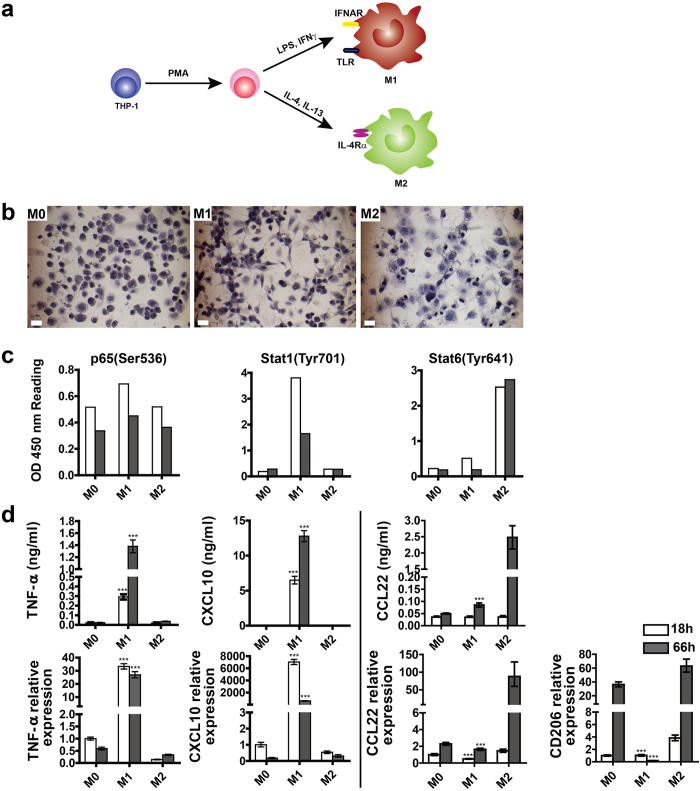
Characterization of THP-1 cells polarization into M1 and M2 macrophages. (**a**) Schematic representation of THP-1 differentiation/polarization. (**b**) Hematoxylin and eosin staining of macrophages at 66 h (scale bar = 10 μm). (**c**) Pathscan ELISA of phospho-p65, Stat1, and Stat6. (**d**) Quantification of M1 and M2 markers in M0, M1 and M2 macrophages by ELISA (upper row) and qPCR (lower row) at 18 h (open bars) and 66 h (filled bars). PCR data are normalized to GAPDH and relative to gene expression level of M0 macrophages at 18 h. Data are shown as mean ± S.D. (n = 4) and represent one of the three independent experiments. Statistical differences between M1 and M2 macrophages were determined by two-tailed Student’s t-test, ***p < 0.001.

A M1 and M2 macrophage polarization model was established using the human monocytic cell line THP-1 (Fig. 1). M1- and M2-polarized macrophages were generated by treating THP-1 cells with PMA and polarizing the cells with LPS/IFN-γ and IL-4/IL-13, respectively^20^. Non-stimulated macrophages M0 served as controls. M1 macrophages adopted an elongated, spindle-shaped cell morphology, whereas M2 macrophages showed flattened, rounded shape with elongated filopodia (Fig. 1b). Pathscan ELISA was used to detect the activation of M1- and M2- related pathways. M1 polarization is dominated by NF-κB and STAT1 pathways, which regulate the expression of a large number of inflammatory genes including TNF-α, CXCL10, IL1β and IL-12 . In contrast, a predominance of STAT-6 activation promotes M2 macrophage polarization, resulting in immunosuppression^21^,^22^. A significant induction of phospho-NF-κB p65 (Ser536) and phospho-Stat1 (Tyr701) was detected in M1 macrophages here at 18 and 66 h (Fig. 1c), indicating the activation of LPS/IFN-γ-stimulated NF-κB and Stat1 pathways. The elevated level of Stat6 (Tyr641) in M2 macrophages suggested the activation of IL-4/IL-13-stimulated STAT6 pathway. We then used PCR and ELISA to assess the M1 (TNF-α, CXCL10) and M2 markers (CCL22, CD206) during macrophage polarization (Fig. 1d). Production of TNF-α and CXCL10 was significantly increased in M1 macrophages, while drastically reduced in M2 macrophages. TNF-α and CXCL10 genes were highly expressed in M1 macrophages and remained low in M2 macrophages. Secretion of CCL22 and gene expression of CCL22 and CD206 were significantly higher in M2 cells as compared to M1 cells. We isolated primary monocytes from the peripheral blood. The isolated monocytes were then predifferentiated with macrophage colony-stimulating factor (M-CSF) and polarized into M1 and M2 macrophages by LPS/ IFN-γ and IL-4/IL-13, respectively. The secretion of TNF- α and CXCL10 in M1 macrophages was significantly higher than M2 macrophages, while the secretion of CCL22 in M2 macrophages was significantly higher than M1 (Supplementary Fig. 1). This pattern is in line with that of THP-1 derived macrophages. Taken together, these data show that THP-1 cells are a useful model for macrophage polarization upon M1 (classic) and M2 (alternative) activation.

### Expression of K_ATP_ channel subunits in polarized macrophages

K_ATP_ channels are versatile targets for control of bioelectric cell state and tissue outcomes^23^,^24^. Although the presence of K_ATP_ channels has been reported in murine macrophage cell lines, such as RAW264.7 and BV2^25^,^26^, there is little evidence for the expression of K_ATP_ channels in human macrophages of different phenotype. K_ATP_ channels are composed of four pore-forming inwardly rectifying K_ir_6.x subunits (K_ir_6.1 or K_ir_6.2), and four regulatory sulfonylurea receptor (SUR1, SUR2) subunits^27^. Different combinations of K_ATP_ subunits compromise various K_ATP_ channels in native tissue with distinct electrophysiological properties and pharmacological sensitivities. Glibenclamide can non-selectively bind to SUR1 or/and SUR2 subunits, while pinaidil selectively binds to SUR2 subunits^27^,^28^.

**Figure 2:**
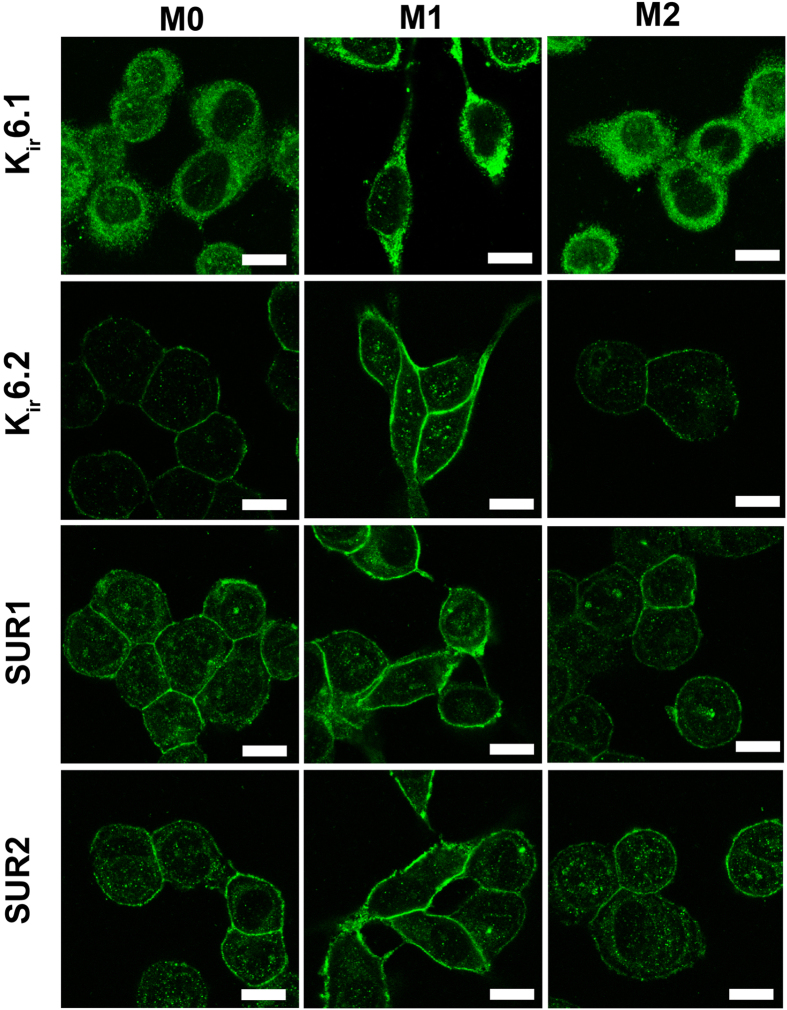
Expression of K_ATP_ channel subunits in macrophages. Immunocytochemistry was performed with specific antibodies against human K_ATP_ subunits K_ir_6.1, K_ir_6.2, SUR1 and SUR2 (scale bar = 10 μm). M0, M1 and M2 macrophages stained positive for all four K_ATP_ subunits. Secondary antibody alone was used as a negative control for each primary antibody. No unspecific antibody binding was detected.

In order to gain insight into the function of K_ATP_ in macrophage polarization, we characterized K_ATP_ subunits in differentially polarized human macrophages. Immunocytochemistry analysis revealed that K_ir_6.1 subunit was localized mostly in the cytosol and Kir6.2 subunit mainly in plasma membranes (Fig. 2). SUR1 and SUR2 subunits staining showed a more intense pattern in plasma membrane, and a diffuse and less intense pattern in cytosol. Moreover, M1 macrophages exhibited enhanced K_ir_6.2, SUR1 and SUR2 expression in cell plasma membrane as compared to M0 and M2 macrophages. This enhancement of the expression of K_ATP_ channel subunits K_ir_6.1, K_ir_6.2, SUR1was also observed in BV2 cells activated with LPS/IFN-γ^26^.Thus, components of K_ATP_ are present in macrophages with a cellular localization consistent with channel function. These subunits might form functional binding sites for drugs such as glibenclamide and pinacidil, facilitating the control of macrophage V_mem_ without the need of gene therapy with exogenous channels.

### Modulation of membrane potential during macrophage polarization

**Figure 3:**
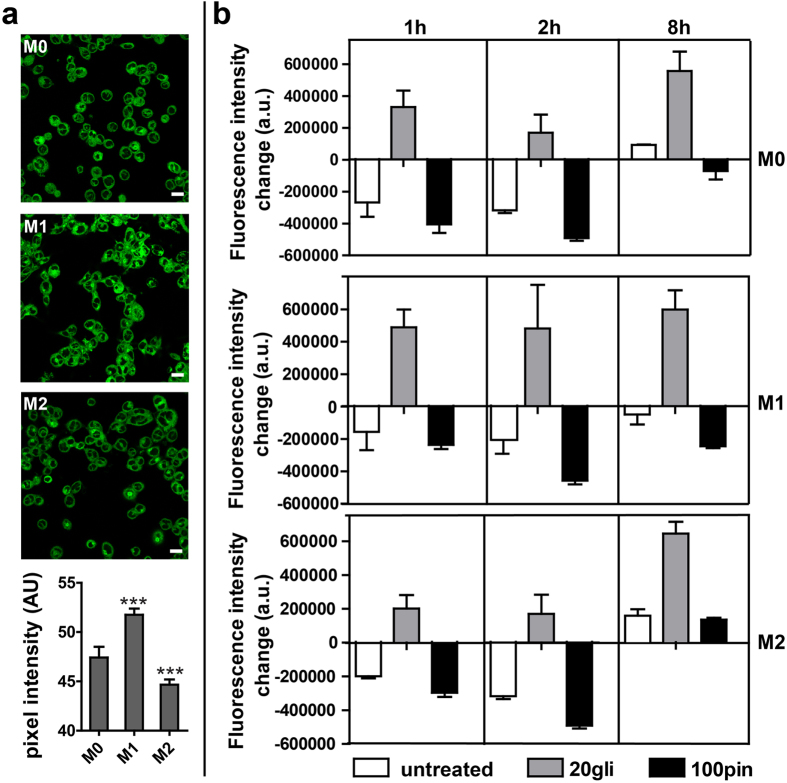
Modulation of membrane potential during macrophage polarization. (**a**) Fluorescence images of macrophages incubated with DIBAC after 18 hours of polarization (scale = 20 μm). Fluorescent intensities of differently polarized macrophages were quantified by Matlab software. Data are represented as mean pixel intensity ± S. D. (N = 7–10 cell fields). Marked samples are statistically different relative to M0 18 h sample (***p < 0.001), as determined by two-tailed Student’s t-test. (**b**) Change of fluorescent intensity after glibenclamide and pinacidil treatment. Data are shown as mean ± S. D. (n = 5) and are representative of two independent experiments.

Membrane potential was tracked to determine whether macrophage V_mem_ changes as a function of cell polarization states. At 18 h, M1 and M2 polarized macrophages showed increased (depolarization) and decreased (hyperpolarization) V_mem_ as compared to M0 macrophages, respectively (Fig. 3a). Changes in membrane potential are among the earliest detectable events in some macrophage functional states, such as classical activation and phagocytosis^29^. In classically activated macrophages, cells undergo a V_mem_ depolarization upon LPS/IFN-γ stimulation accompanying by a predominance of outward current^16^. We observed that LPS/IFN stimulation caused depolarized V_mem_ at 18 h, which is consistent with some previous reports. For example, the V_mem_ of LPS activated murine macrophage-like J774.A1 cells depolarized by 40.7 ± 17.9% as compared to non-activated macrophages along with increased outward current and decreased I_Kir_^29^. Similarly, an increase in I_Kout_ and reduction in I_Kir_ was detected in mouse bone marrow macrophages after LPS activation^17^.

The intrinsic difference in membrane potential in macrophages of different phenotype may imply that modulation of V_mem_ by selective activation/inhibition of specific potassium current/ion channel can regulate macrophage differentiation/polarization. We next studied the change of V_mem_ in macrophages upon blocking and opening of K_ATP_ channels by glibenclamide and pinacidil. An increase in fluorescence intensity was induced by glibenclamide treatment, indicating a depolarization effect (Fig. 3b). In contrast, pinacidil caused cell hyperpolarization, except that a depolarizing effect was observed in M2 macrophages at 8 h. These results indicated that glibenclamide and pinacidil treatments effectively changed macrophage V_mem_ and V_mem_ in macrophages is a function of K_ATP_ channels. These results indicated that glibenclamide and pinacidil treatments effectively changed macrophage V_mem_ and V_mem_ in macrophages is a function of K_ATP_ channels.

### Effects of glibenclamide and pinacidil prepolarization treatment on macrophage polarization

**Figure 4:**
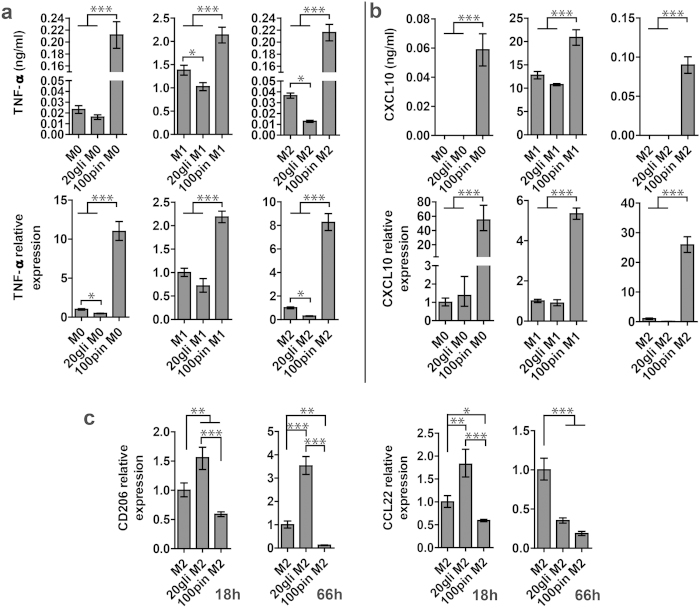
Effects of glibenclamide and pinacicil prepolarization treatment on macrophage polarization. (**a**) TNF-α and (**b**) CXCL10 secretion (upper row) and gene expression (lower row) in M0, M1 and M2 macrophages at 66 h. (**c**) CD206 and CCL22 gene expression in M2 macrophages at 18 and 66 h. PCR data are normalized to GAPDH and relative to gene expression level of untreated controls. The data are shown as mean ± S. D (n = 3–4) and representative of two independent experiments. Statistical significances are reported among samples at the same time point using one way Anova (***p < 0.001, **p < 0.01 and *p < 0.05).

To determine whether V_mem_ modulation allows functional control of macrophage polarization, 20 μM glibenclamide and 100 μM pinacidil were added before introducing LPS/IFN- γ or IL-4/IL-13 (prepolarization treatment). No apparent cytotoxic effects of glibenclamide and pinacidil were observed at the concentration used (Supplementary Fig. 2). In M1 macrophages, glibenclamide significantly suppressed the secretion and gene transcript of TNF-α (Fig. 4a), while pinacidil enhanced the production and gene expression of both TNF-α and CXCL10 (Fig. 4a,b). Notably, in M0 and M2 macrophages, pinacidil treatment increased secretion and gene expression of TNF-α and CXCL10 markedly. In M2 macrophages, glibenclamide and pinacidil exposure caused significant upregulation and downregualtion of CD206 expression, respectively (Fig. 4c). Together, pinacidil treatment enhanced M1 markers and suppressed M2 markers, suggesting a proinflammatory effect. While glibenclamide treatment reduced M1 marker (TNF-α) in M1 macrophages, it augmented M2 marker (CD206) expression in M2 macrophages and demonstrated an anti-inflammatory effect.

In macrophages, prior activation of a number of K^+^ channels, such as K_v_ and BKCa, is involved in the release of LPS-induced immune mediators^18^,^30^,^31^,^32^, which is associated with M1 polarization. However, there are no studies on the role of membrane electric potential in macrophage polarization toward M2 phenotype. The K_ATP_ channel is a well-known metabolic sensor that couples cell metabolism with transmembrane potassium fluxes in many cell types^28^,^33^. Recent data suggested that macrophages adopted distinct metabolic features that regulate their functional polarization. M1 macrophages make use of an anaerobic glycolytic pathway, while M2 macrophages adopt an oxidative glucose metabolism (fatty acid oxidation) pathway^34^. We reasoned that bioelectric modulation targeting the K_ATP_ channel might affect macrophage phenotype. In this study, using glibenclamide (K_ATP_ channel blocker) and pinacidil (K_ATP_ channel opener), we aimed to study the effect of V_mem_ modulation on macrophage polarization toward M1 and M2 phenotype.

We found that glibenclamide treatment had an anti-inflammatory effect by suppressing TNF-α release and gene expression in M1 macrophages. Consistent with our findings, recent studies reported that glibenclamide had anti-inflammatory effect in some diseases. For example, glibenclamide could rescue the progression of atherosclerosis in mice and reduced mortality in melioidosis was found in patients taking glibenclamide^25^,^35^. An *in vitro* study using RAW264.7 cells showed that glibenclamide significantly inhibited production of TNF-α induced by LPS^25^. It is particularly interesting that glibenclamide also enhanced the expression of M2 marker CD206 in M2 polarized macrophages, but not in M0 and M1 polarized macrophages.

We also found that pinacidil had proinflammatory effects on M0, M1 and M2 macrophages, and pinacidil was able to induce macrophage polarization toward M1 phenotype without LPS/IFN-γ. Pinacidil is a well-known selective K_ATP_ opener, and it has very little effect on other ion channels^36^. The distinct effect of pinacidil in our study suggested that pinacidil could activate K_ATP_ channels during macrophage polarization, and the resulting membrane hyperpolarization could affect M1 marker-related pathways and augment M1 markers. More importantly, the discovery that M1 polarization can not only be suppressed by glibenclamide, but can also be augmented with pinacidil, suggesting that V_mem_ acted as an instructive signal for macrophage polarization. A similar effect was reported in our previous study of hMSCs^7^, in which depolarization inhibited and hyperpolarization enhanced osteogenic differentiation of hMSCs.

### Effects of glibenclamide and pinacidil treatment on prepolarized macrophages

**Figure 5:**
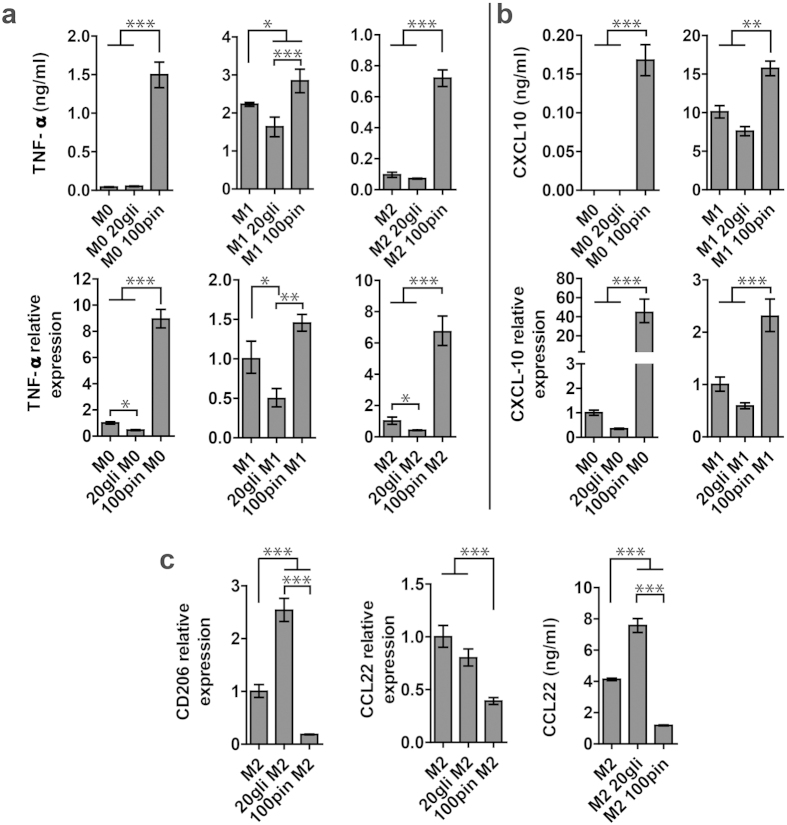
Effects of glibenclamide and pinacidil treatment on prepolarized macrophages. (**a**) TNF-α release into supernatant (upper row) and gene expression (lower row). (**b**) CXCL10 release (upper row) and gene expression (lower row). (**c**) CD206 gene expression, CCL22 secretion and gene expression in prepolarized M2 macrophages. PCR data are normalized to GAPDH and relative to gene expression level of untreated samples. Data are shown as mean ± S. D. (n = 3–4) and are representative of two independent experiments. Statistical significance are determined by one way Anova (***p < 0.001, **p < 0.01 and *p < 0.05).

We next studied whether glibenclamide and pinacidil treatment affects the polarization states of macrophages after they have been prepolarized, which could indicate that V_mem_ plays a role not only during the polarization process, but also in the maintenance of macrophage phenotype. Macrophages were prepolarized to M0, M1 and M2 macrophages for 18 hours and subsequently subjected to glibenclamide and pinacidil treatment for 48 hours by adding 20 μM glibenclamide and 100 μM pinacidil into culture medium. In consistent with our findings in the prepolarization treatment study, prepolarized M0, M1 and M2 macrophages exposed to pinacidil all had higher levels of M1 markers and lower M2 markers compared to the untreated cells (Fig. 5). Moreover, glibenclamide treatment downregulated the gene expression of M1 markers. In prepolarized M2 macrophages, glibenclamide treatment upregulated CD206 gene expression and enhanced CCL22 secretion without affecting its gene expression (Fig. 5c).

**Figure 6:**
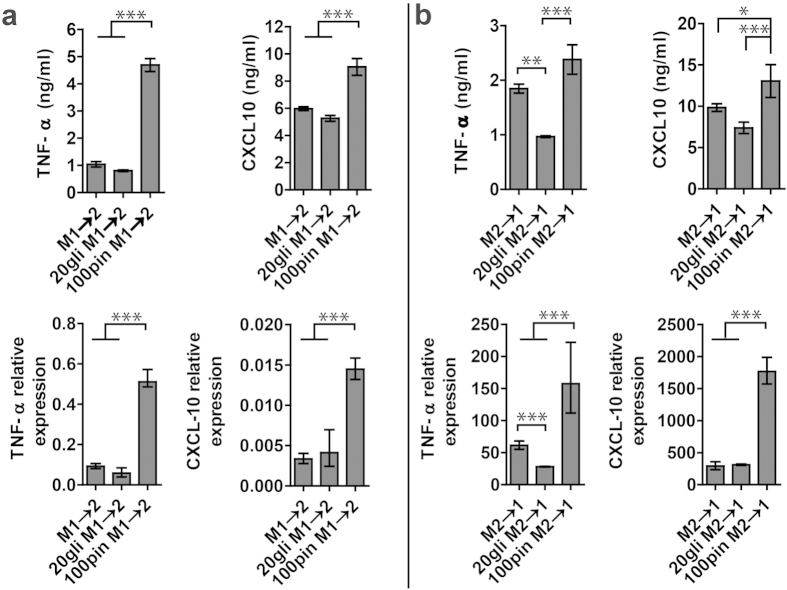
Effects of glibenclamide and pinacidil treatment on macrophage plasticity: quantification of M1 markers (66 h) by ELISA and qPCR. (**a**) Release and gene expression of TNF-α and CXCL10 during M1 to M2 transdifferentiation. (**b**) Release and gene expression of TNF-α and CXCL10 during M2 to M1 transdifferentiation. M1 to M2 PCR data are normalized to GAPDH and relative to gene expression of M1 at 18 h. M2 to M1 PCR data are normalized to GAPDH and relative to gene expression of M2 at 18 h. Data are shown as mean ± S. D. (n = 3–4) and are representative of two independent experiments. Statistical significance are reported among samples at 66 h using one way Anova (***p < 0.001 and *p < 0.05).

In line with our findings in the prepolarization treatment study, glibenclamide and pinacidil also exerted opposite control over the phenotype of predifferentiated/polarized macrophages. More importantly, this effect was observed in all three types of macrophages as demonstrated by the gene expression data (Fig. 6), and this effect was only found in M1 macrophages in the prepolarization treatment study. One possible explanation for this difference could be that predifferentiated/polarized macrophages presented an expression pattern of K_ATP_ subunits that facilitated more efficient glibenclamide and pinacidil binding.

The anti-inflammatory effect of glibenclamide and pro-inflammatory effect of pinacidil on macrophage polarization suggested that K_ATP_ channel are potential targets for immunomodulation (Supplementary Fig. 2). Calcium plays a determinant role in the generation of proinflammatory responses and voltage-gated calcium channels (VGCC) have been identified in mouse peritoneal macrophage and human peripheral blood mononuclear cell^37^,^38^. Binding of glibenclamide/pinacidil to K_ATP_ channels induced depolarization/hyperpolarization of macrophage plasma membrane. The resulting V_mem_ change might affect the intracellular Ca^2+^ concentration through VGCCs in cell membrane, hence mediate the signaling pathways responsible for the expression and release of cytokine/chemokine. NMDA receptors (NMDARs) have been shown to be upstream of K_ATP_ channels in specific neurons^39^,^40^ and pancreatic islets^41^ and the activated K_ATP_ channels participate in the downstream signaling events. The presence of NMDARs in some macrophages opens the possibility to control macrophage polarization by targeting NMDARs.We studies the effect of two NMDA receptor antagonists, MK-801 and dextrophan (DXO), on the polarization of macrophage toward M1 phenotype. It was found that both MK-801 and DXO suppressed the secretion and gene transcript of TNF-α (Supplementary Fig. S4), suggesting that MK-801 and DXO may phenocopy the effect the glibenclamide. Thus, the antagonists of NMDA receptors may also be useful for the treatment of diseases featured by M1 macrophage - mediated inflammatory processes.

### Effects of glibenclamide and pinacidil treatment on macrophage plasticity

Plasticity and diversity are hallmarks of cells of the monocyte-macrophage lineage. Polarized macrophage can be switched, to some extent, from one functional phenotype to another in response to microenvironmental signals of the local milieu^42^,^43^. We therefore asked the question whether V_mem_ modulation can affect the transdifferentiation between M1 and M2 phenotypes. Macrophages were polarized to M1 and M2 macrophages for 18 hours and subsequently transdifferentiated toward M2 and M1 macrophages for 48 hours, respectively. Glibencalmide and pinacidil were supplemented to culture medium 10 minutes before the addition of M1 and M2 stimuli for trandifferentiation. IL-4/IL-13 stimulation of prepolarized M1 macrophages reduced M1 markers and increased M2 markers, resulting in an intermediate macrophage phenotype unbalanced toward M1 (Fig. 4). On the other hand, LPS/IFN-γ stimulation of prepolarized M2 macrophages induced a M1-like phenotype with high levels of M1 markers and low levels of M2 markers. Thus we demonstrated the ability of polarized THP-1 macrophages to respond to secondary stimuli and even to revert their functional state. Pinacidil inhibited the M1→2 transdifferentiation by enhancing M1 markers and suppressing M2 markers, while glibenclamide did not cause significant effect (Fig. 4A, Supplementary Fig. 3). During M2→1 transdifferentiation, glibenclamide inhibited TNF-α secretion and gene expression and did not cause significant changes in CXCL10 secretion and gene expression. Pinacidil promoted the transdifferention from M2 to M1 phenotype with increased M1 markers and reduced M2 markers (Fig. 4A, Supplementary Fig. 3).

The results revealed that M1 and M2 macrophages retained their ability to transdifferentiate toward M2 and M1 phenotype in the presence of glibenclamide and pinacidil, indicating that macrophage plasticity were preserved during V_mem_ modulation and that V_mem_ modulation could be a useful tool to fine-tune macrophage plasticity.

Immune response to an implant involves all types of macrophages, with M1-like proinflammatory macrophages in the early phase followed by M2-like regulatory and wound healing macrophages in the later resolution stage^44^,^45^. Current biomaterial designs focus on developing materials that preferentially induce M2 macrophage polarization, whereas little attention has been paid to proinflammatory M1 macrophages^46^,^47^,^48^,^49^,^50^,^51^. Macrophages adopt different phenotypes during different stages and secrete various types of cytokines, chemokines, and growth factors^52^,^53^. There is increasing evidence that inflammatory macrophages (M1) play an important role in the early phase of bone fracture repair in both human and animals^54^,^55^. Proinflammatory cytokines that are generally associated with M1 responses, such as TNF-α, IL-1β and IL-6, have been detected during this early stage. These cytokine have also been shown to be able to promote osteogenesis of progenitor cells *in vitro* and *in vivo*^55^,^56^. Thus, temporal delivery of V_mem_ -modulating chemicals during diverse phases of tissue regeneration may offer a promising way to regulate macrophage phenotype for better outcomes.

The skewed polarization of macrophages is also found to be closely associated with many pathological conditions, such as cancer and autoimmune diseases. Tumor associated macrophages (TAMs) generally display an M2-like phenotype with tumor-promoting function, in contrast to the proinflammatory and anti-cancer function of M1 macrophages^21^. We co-cultured human lung cancer cell line A549 with glibenclamide/pinacidil treated M0, M1 and M2 macrophages. The invasion assay of A549 cells showed that M1 macrophages inhibited A549 invasion ability compared to M0 macrophages, while M2 macrophages increased A549 invasion ability. Moreover, for both M1 and M2 macrophages, pinacidil decreased A549 invasion ability, while glibenclamide treatment increased A549 invasion ability (Supplementary Fig. S6). These results suggest that the V_mem_ modulation of macrophage polarization toward tumor-killing phenotype might provide a useful tool for macrophage-centered anticancer therapy. Inflammatory macrophages have been implicated in the pathogenesis of many autoimmune diseases, such as multiple sclerosis and rheumatoid arthritis^57^. These macrophages produce inflammatory cytokines which are the important drivers of autoimmune inflammation. Thus, the functional skewing of macrophage phenotype toward anti-inflammatory phenotype by V_mem_ modulation may be a new therapeutic approach in the control of autoimmune disease.

## Conclusions

K_ATP_ channels were found to play a vital role in the modulation of macrophage polarization. Modulation of cell membrane electric potential by targeting K_ATP_ channels not only affects macrophage differentiation/polarization, but also influences the phenotype of prepolarized macrophages. Furthermore, this biophysical feature can fine-tune macrophage plasticity. Blockage of K_ATP_ channel by glibenclamide decreases the secretion and gene expression of selected M1 markers, while opening K_ATP_ channel by pinacidil augments M1 markers. More interestingly, glibenclamide could promote macrophage alternative activation by enhancing M2 marker CD206 during M2 polarization. These data suggested that control of bioelectric properties of macrophages, using small molecule drugs already approved for human use, could offer a promising way to regulate macrophage phenotype, hence providing a useful tool in regenerative medicine. Future work will allow fine-tuning of these bioelectrical factors to optimize the immunomodulating activity of biomaterials.

## Additional Information

**How to cite this article**: Li, C. *et al.* Bioelectric modulation of macrophage polarization. *Sci. Rep.* **6**, 21044; doi: 10.1038/srep21044 (2016).

## Supplementary Material

Supplementary Information
