# Supplementary material for: Bioelectric modulation of macrophage polarization
Source: Sci Rep. 2016 Feb 12;6:21044. doi: 10.1038/srep21044 (PMC4751571; doi:10.1038/srep21044)
Supplement: Supplementary Information [file srep21044-s1.pdf]

Supplementary Information

Bioelectric modulation of macrophage polarization

Chunmei Li, Michael Levin, David L. Kaplan*

Chunmei Li

Department of Biomedical Engineering, Tufts University, 4 Colby Street, Medford, MA 02155
Email address: chunmei.li@tufts.edu
Phone number: 6176272581

Michael Levin

Department of Biology, and Center for Regenerative and Developmental Biology, Tufts University, Suite 4600, 200 Boston Avenue, Medford, MA 02155, USA
Email address: michael.levin@tufts.edu
Phone number: 6176276161

David L. Kaplan

Department of Biomedical Engineering, Tufts University, 4 Colby Street, Medford, MA 02155
Email address: david.kaplan@tufts.edu
Phone number: 6176273251

Supplementary Figures S1-S3

Supplementary methods

Monocyte isolation, differentiation and polarization of human macrophages Human monocytes were purified from whole blood (Research Blood Components, LLC, MA). Peripheral blood mononuclear cells (PBMCs) were first obtained from the whole blood using ficoll gradient centrifugation. The CD14+ monocytes were isolated from the PBMCs by positive selection using magnetic CD14 MicroBeads (Miltenyi Biotech, Germany) according to the manufacturer's instructions. Purities of monocyte preparations were analyzed by flow cytometry using fluorescein isothiocyanate-conjugated mAb with specificity and viability of the isolated cells using 7-amino-actinomycin (7-AAD) staining. Flow cytometric analyses were performed in a FACSCalibur flow cytometer (BD Biosciences, Germany) and data were analyzed by FlowJo software (FlowJo, US).

The isolated monocytes were then differentiated into macrophages by 50 ng/ml M-CSF for 6 days in RPMI 1640 medium supplemented with 10% FBS, 100 U/ml penicillin and 100 mg/ml streptomycin antibiotics. For M1 polarization, macrophages were exposed to medium containing 100 ng/ml LPS and 20 ng/ml IFN-γ. For M2 polarization, macrophages were exposed to medium containing 20ng/ml IL-4 and 20ng/ml IL-13.

Effect of MK-801 and dextrophan (DXO) Macrophages were prepolarized to M0, M1 and M2 macrophages for 18 hours and subsequently subjected to MK-801 and DXO treatment for 48 hours by adding MK-801 (200 μM) and DXO (200 μM) into culture medium.

THP-1 and A549 co-culture The human lung adenocarcinoma cell line A549 was obtained from ATCC and maintained in RPMI 1640 medium (Invitrogen, CA) supplemented with 10% heat-inactivated FCS, 100 U/ml penicillin and 100 mg/ml streptomycin. The THP-1 derived macrophages and A4549 cells were co-cultured using 0.4 μm Transwell cell culture insert (Corning, US). The THP-1 cells (1×10^6 cells/well) were seeded in the upper inserts of a six-well plate and predifferentiated/prepolarized to M0, M1 and M2 macrophages as described above. The prepolarized cells were then treated with glibelclamide or pinacidil for 24 hours. The A549 cells were seeded in the six-well companion plates (2.5×10^5 cells/well) and allowed to attach for 24 hours. The inserts with THP-1 cells were then placed in plates containing A-549 cells and the co-culture systems were incubated for 24 hours in serum-free RPMI 1640.

Invasion assays The invasion capability of A549 cells co-cultured with THP-1 derived macrophages was conducted using Matrigel invasion chamber (24 well, 8 μm pore size, Corning, US). After 24 hours of co-culture with THP-1 derives macrophages, A-549 cells were collected and dispersed in serum-free RPMI 1640 medium with 0.1% BSA. Two hundred microliter of cell suspension (2.5×10^4 cells) was added to the upper inserts, and 500 μl of RPMI 1640 supplemented with 10% FBS was added to the lower chamber. After incubation for 24 hours, the invaded A549 cells were fixed with 4% PFA and stained with DAPI. Data is expressed as the percent invasion through Matrigel coated membrane relative to the migration through the uncoated control membrane.

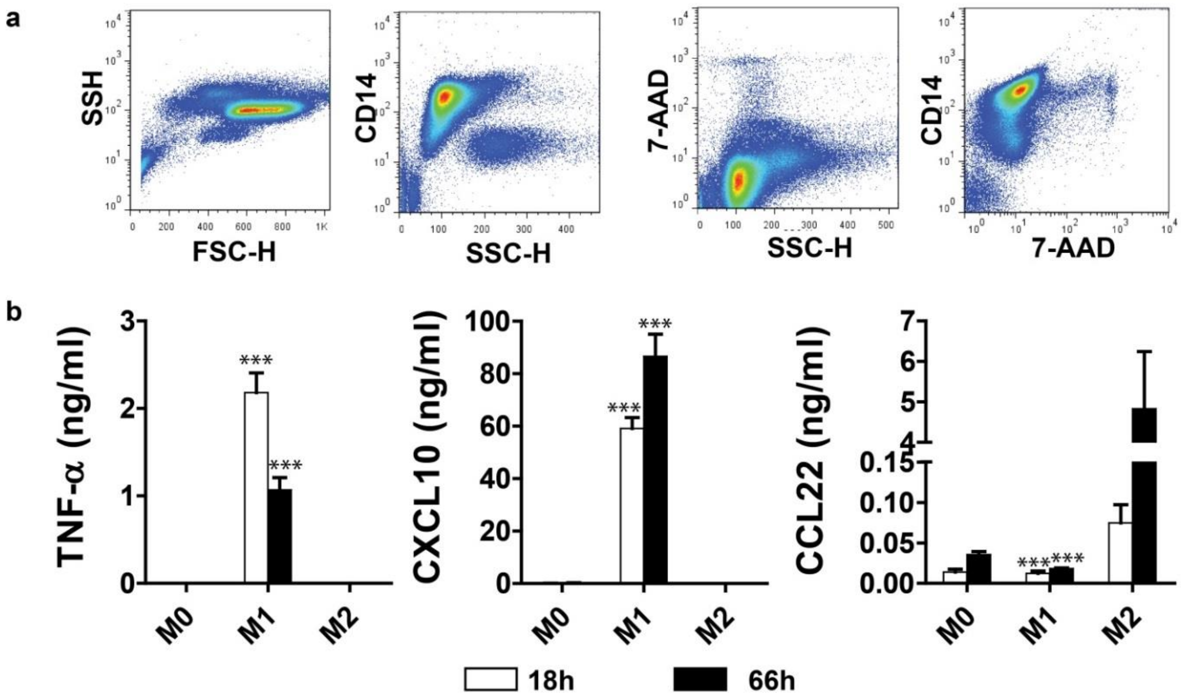

Supplementary Figure S1. Primary monocyte-derived macrophages were obtained by monocytes treated with 50 ng/ml M-CSF or 50 ng/ml GM-CSF for 7 days. Purity of monocytes after MACS separation (CD14+) was above 95% (a). Quantification of TNF-α, CXCL10 and CCL22 in M0, M1 and M2 macrophages by ELISA at 18h (open bars) and 66h (filled bars) (b). Data were represented as mean ± S.D. (N = 3-4). Statistical differences between M1 and M2 macrophages were determined by two-tailed Student's t-test, ***$p < 0.001$.

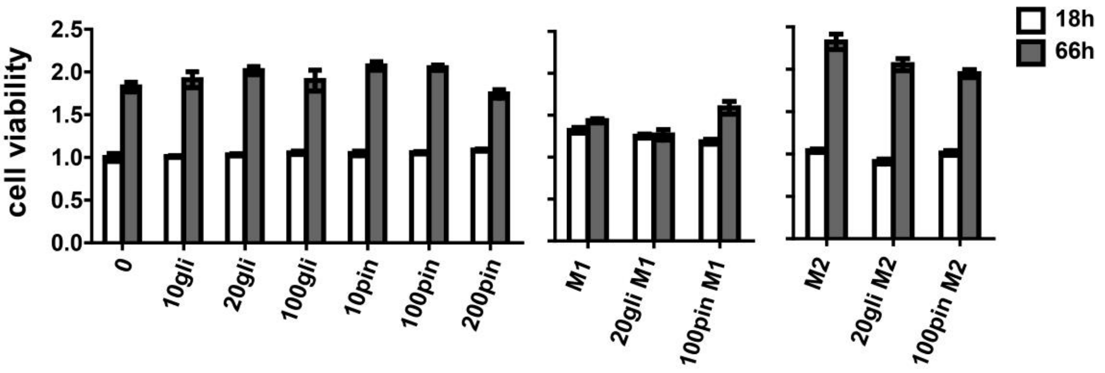

Supplementary Figure S2. Cytotoxicity of glibenclamide and pinacidil on differentially polarized macrophages. Cells were seeded in 24-well plates at a density of 250,000 cells/well and cell viability was determined by Alamar blue assay. No apparent cytotoxicity of glibenclamide (10, 20 and 100 μM) and pinacidil (10, 100 and 200 μM) was observed.

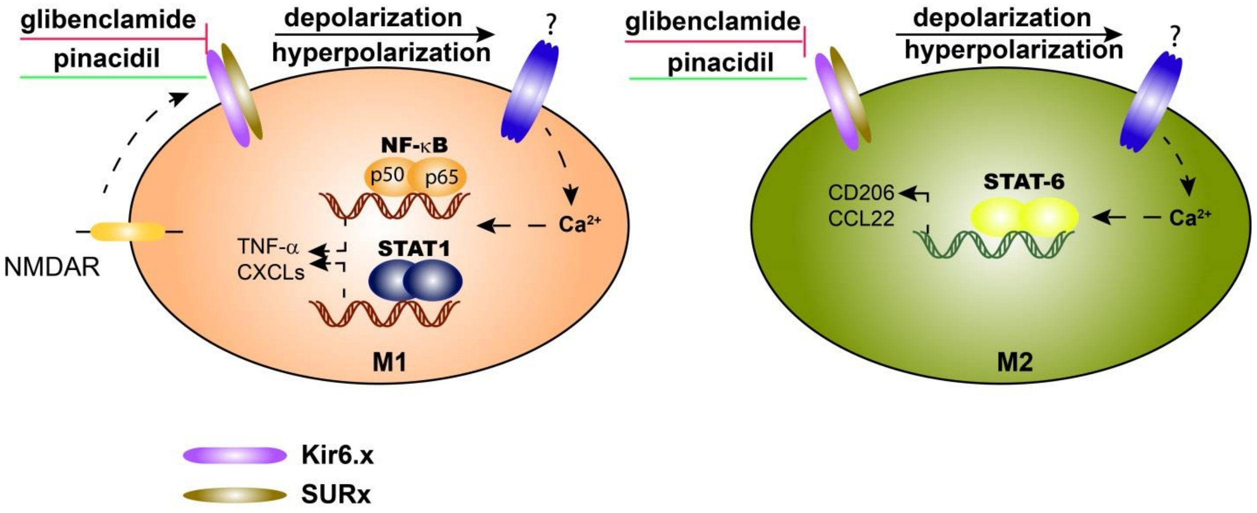

Supplementary Figure S3. Schematic representation of the effects of glibenclamide and pinacidil treatment on macrophage polarization Glibenclamide and pinacidil caused depolarization and hyperpolarization in macrophage plasma membrane, respectively. This V_{mem} modulation might affect intracellular Ca^{2+} level, which mediates the related pathways controlling secretion of proinflammatory and anti-inflammatory cytokine/chemokines. Unclear signal transduction is indicted by the dashed arrows.

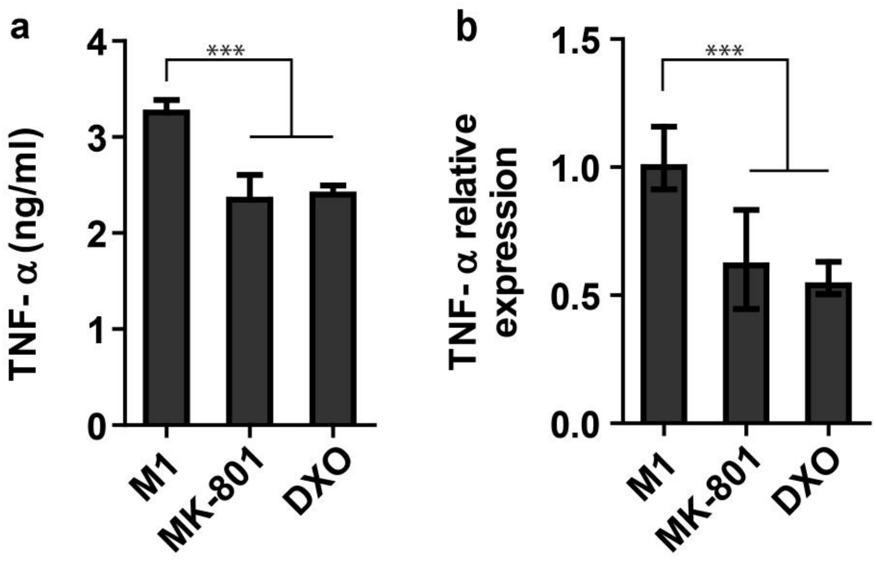

Supplementary Figure S4. **MK-801 and dextrorphan (DOX) treatment inhibited the secretion (a) and gene expression (b) of TNF-α.** PCR data are normalized to GAPDH and relative to gene expression level at 18h. Statistical significance are reported among samples at 66h, *** $p < 0.001$.

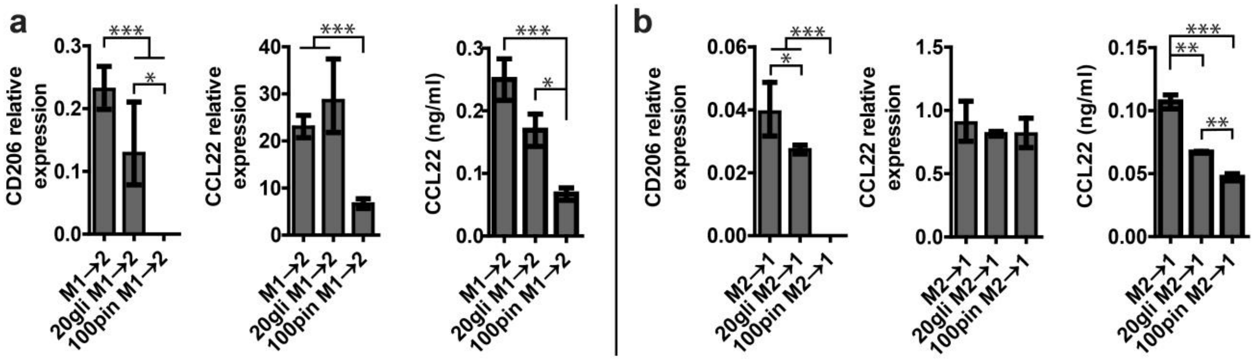

Supplementary Figure S5. **Effects of glibenclamide and pinacidil treatment on macrophage plasticity**: quantification of M2 markers (66h) by ELISA and qPCR. Release of CCL22 and gene expression of CCL22 and CD206 during (**a**) M1 to M2 and (**b**) M2 to M1 transdifferentiation. PCR data are normalized to GAPDH and relative to gene expression level at 18h. Statistical significance are reported among samples at 66h, *** p < 0.001, ** p < 0.01, and * p < 0.05.

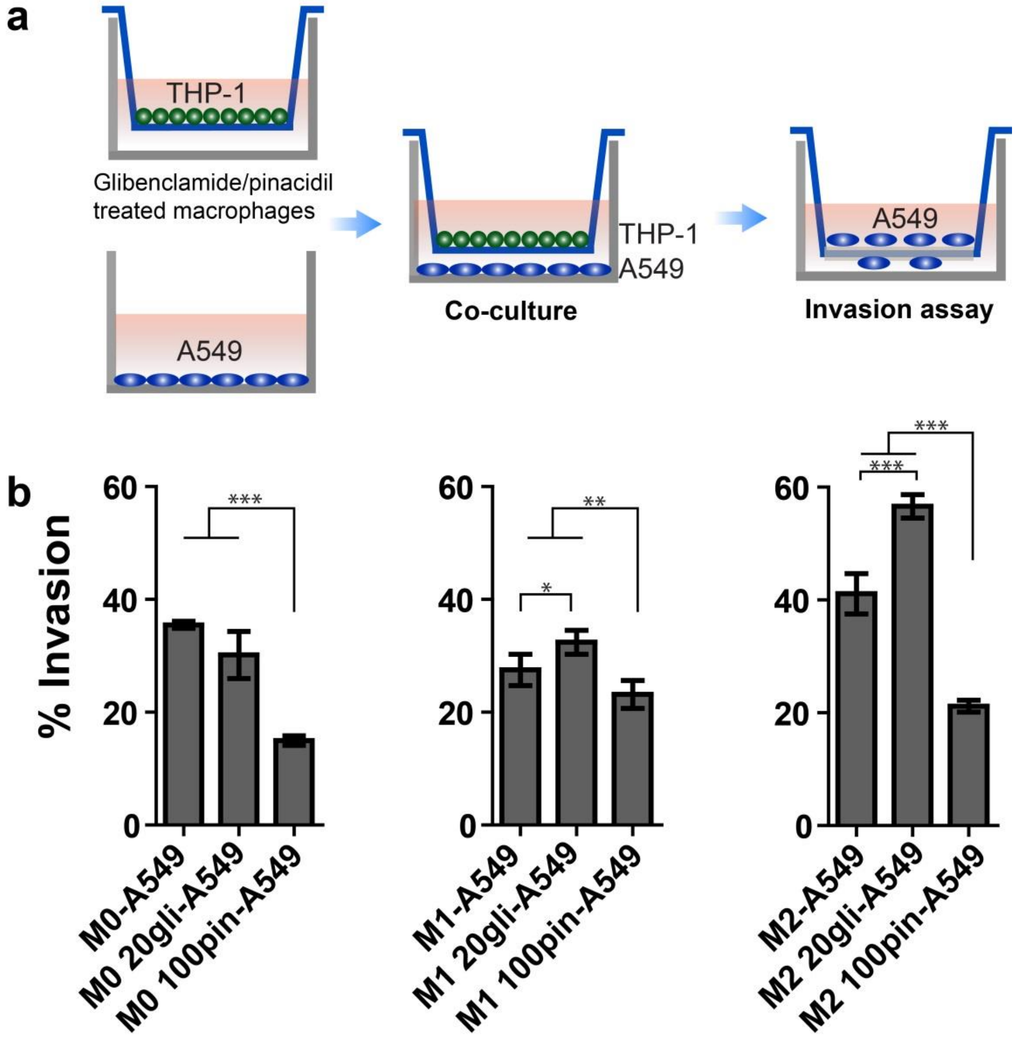

Supplementary Figure S6. Effect of glibenclamide/pinacidil treatment of macrophages on cancer cell invasion. (a) Glibenclamide/pinacidil treated macrophages were co-cultured in indirect contact with A549 cells. (b) Invasion ability of A549 cells after co-culturing with different types of macrophages. Data were represented as mean ± S.D. (N = 3-4). Data are shown as mean ± S. D. (n =3-4) and are representative of two independent experiments. Statistical significance are determined by one way Anova (*** $p < 0.001$, ** $p < 0.01$, and * $p < 0.05$).
